# Rectangular method: a modified technique for sampling the ischemic border zone in a rat model of transient middle cerebral artery occlusion

**DOI:** 10.1590/1414-431X2023e13140

**Published:** 2023-12-11

**Authors:** Sui-yi Xu, Mao-mei Song, Xue Pan, Shi-na Song, Qi Zhang, Chang-xin Li

**Affiliations:** 1 Department of Neurology, Headache Center, The First Hospital of Shanxi Medical University, Taiyuan, Shanxi, China

**Keywords:** Stroke, Animal model, Middle cerebral artery occlusion

## Abstract

To date, there have been three common methods for sampling the cerebral ischemic border zone in a rat model of transient middle cerebral artery occlusion (tMCAO): the “two o'clock method”, the “diagonal method”, and the “parallel line method”. However, these methods have their own advantages and limitations. Here, we propose a modified technique (the “rectangular method“) for sampling the ischemic border zone. A rat tMCAO model was prepared under the support of a compact small animal anesthesia machine. Cerebral blood flow was monitored by high-resolution laser Doppler to control the quality of modeling, and 2,3,5-triphenyl tetrazolium chloride (TTC) staining was used for cerebral infarction location assessment. Superoxide dismutase 2 (SOD2), cysteinyl aspartate specific proteinase (caspase)-3, caspase-9, and heat shock protein 70 (HSP70) were used to verify the reliability and reproducibility of the rectangular method. The expression of biomarkers (SOD2, caspase-3, caspase-9, and HSP70) in the traditional (two o'clock method after TTC staining) and modified (rectangular method) groups were increased. There were no significant differences between the groups. The rectangular method proposed herein is based on a modification of the diagonal method and parallel line method, which could provide a directly observable infarct borderline and a sufficient sampling area for subsequent experimental operations regardless of the cerebral infarct location. The assessed biomarkers (SOD2, caspase-3, caspase-9, and HSP70) demonstrated the reliability and reproducibility of the rectangular method, which may facilitate inter-laboratory comparisons.

## Introduction

Saving the ischemic penumbra is an eternal topic in the treatment of acute cerebral infarction (1). Studies on ischemic border zone are mostly conducted in animal experiments, among which the rat model of transient middle cerebral artery occlusion (tMCAO) is most commonly used. Of note, some studies do not specify how the ischemic border zone was sampled in the research method section, which may lead to poor reproducibility of the experimental results and greatly affect the credibility of the results.

**Figure 1:**
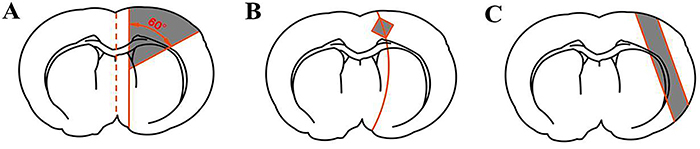
The current three most commonly used methods for sampling cerebral tissue at the ischemic border zone: **A**, Two o'clock method; **B**, Diagonal method; **C**, Parallel line method. Shadows represent the ischemic border zone to be sampled.

To date, three common methods have been used to locate the ischemic border zone. We figuratively refer to the first method as the “two o'clock method”, which was first proposed by Ashwal et al. (2). As shown in Figure 1A, the coronal section at the striatum level was selected to find the midline of the cerebral hemispheres on both sides, and then the ipsilateral side was cut longitudinally approximately 2 mm beside the midline to remove the brain tissue supplied by the anterior cerebral artery. The ischemic border zone was obtained by making a transverse diagonal cut at approximately the two o'clock position. The second method is figuratively referred as the “diagonal method”, which frequently appears in some studies (3,4). As shown in Figure 1B, a square with an area of 1 mm^2^ was made in the coronal section at the level of the striatum stained with 2,3,5-triphenyl tetrazolium chloride (TTC) by taking the boundary between white- and red-dyed tissue as the diagonal, which was defined as the ischemic border zone. Lastly, the third method figuratively termed the “parallel line method” was proposed by Hori et al. (5). As shown in Figure 1C, the post-stained coronal section was identified at the level of the striatum and the boundary between white- and red-stained tissues was confirmed; a line was then drawn equidistant and parallel to the boundary. The area between this line and the boundary was defined as the ischemic border zone.

All three methods have their own limitations. First, the traditional two o 'clock method is not performed under the guidance of TTC staining. As known, infarct areas are susceptible to a variety of objective factors, such as the weight of the rat, the diameter and the insertion depth of the filament, and vascular variations in rats that may lead to a shift in infarct boundaries. If the tissues are taken blindly in the two o'clock direction, the reproducibility of the experiment may be affected. Second, the area of the square obtained by the diagonal method is 1 mm^2^, which is too small to extract enough target proteins to meet the requirements of western blots. Moreover, the parallel line method may only be applicable to cortical infarcts. To overcome these limitations, we propose a modified technique (named the “rectangular method”) for sampling the ischemic border zone in a rat tMCAO model.

Is the ischemic border zone obtained by the modified rectangular method as representative as that obtained by the traditional two o'clock method? A literature search revealed that there are several molecular markers of the ischemic border zone that are generally recognized as present: SOD2 is a mitochondrial superoxide dismutase with the function of a superoxide anion scavenger (6). SOD2 plays an important role in the antioxidant defense against reactive oxygen species-mediated ischemic brain injury and is significantly expressed in the ischemic border zone (6-8). Apoptosis plays an important role in neuronal death in the ischemic border zone (9), with caspase-3 being a key mediator in the caspase-dependent apoptosis pathway and caspase-9 its upstream (10). In the caspase-dependent apoptosis pathway, caspase-3 and caspase-9 are activated, resulting in significant expression in the ischemic border zone (11). HSP70 plays an important role in early brain protection in acute focal cerebral ischemia, as demonstrated by experiments with HSP70 gene knockout mice (12). Some studies have suggested that HSP70 is the gold standard for biochemical markers of the ischemic border zone in animal stroke models. Its expression in the ischemic border zone is significantly increased, protecting neurons (13,14). Animal studies have shown that exogenous HSP70 can reduce the level of apoptosis in the ischemic border zone (15). This study intended to compare the protein levels of molecular markers of the ischemic border zone in tissues taken by the rectangular method and the two o’clock method.

## Material and Methods

### Preparation of the rat tMCAO model

The animal study protocol was approved by the Ethics Committee of the Laboratory Animal Center of the First Hospital of Shanxi Medical University (SYXK 2022-0008). Sprague-Dawley male rats were provided by the Experimental Animal Center of Shanxi Medical University. A total of 20 male rats weighing 220±20 g at the age of 6-8 weeks were included in this study. Three rats were excluded during modeling for not meeting quality control of high-resolution laser Doppler. Two rats were accidently sacrificed after modeling and necropsy revealed that one died of subarachnoid hemorrhage and the other died of brain herniation following massive cerebral infarction. The remaining 15 rats were randomly divided into 3 groups: the control group, the traditional group (two o'clock method after TTC staining), and the modified group (rectangular method) (n=15). All rats were housed in a humidity- and temperature-controlled environment with regular light/dark cycles and free access to food and water.

**Figure 2:**
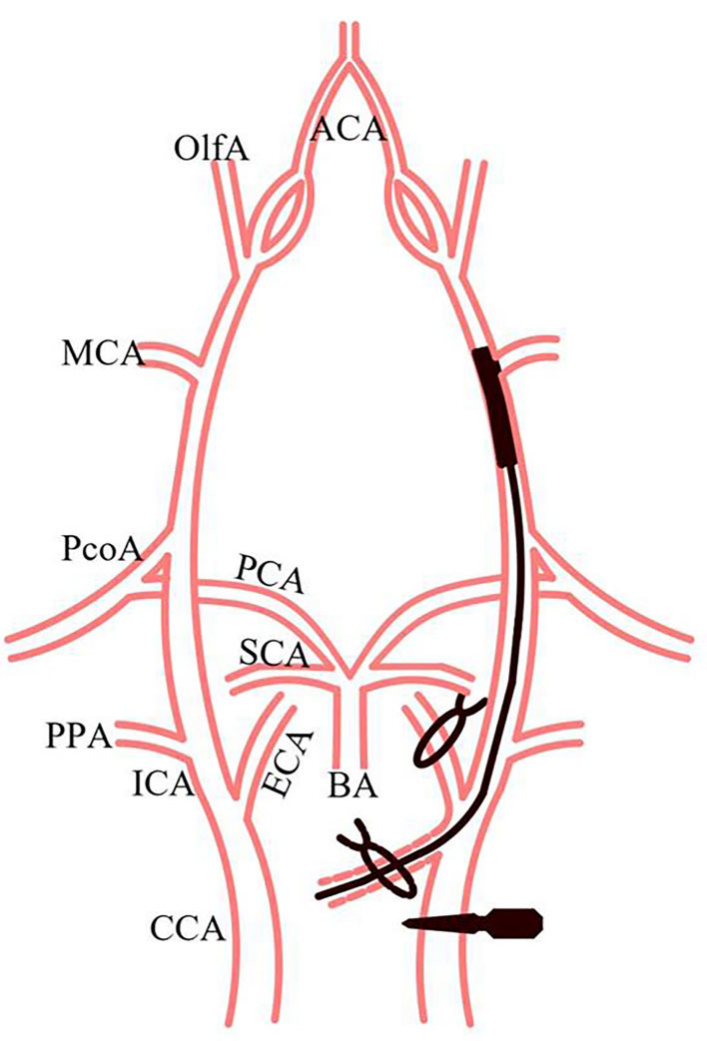
Schematic diagram of a rat transient middle cerebral artery occlusion model operation. ACA: anterior cerebral artery; OlfA: olfactory artery; MCA: middle cerebral artery; PcoA: posterior communicating artery; PCA: posterior cerebral artery; SCA: superior cerebellar artery; PPA: pterygopalatine artery; ECA: external carotid artery; ICA: internal carotid artery; CCA: common carotid artery; BA: basilar artery.

The rat tMCAO model was established according to the method proposed by Longa et al. (16). Briefly, rats were anesthetized with 5% isoflurane using a compact small animal anesthesia machine (R5401E, RWD, China) and then maintained at 1.5-2.5% isoflurane using a face mask. Rats were placed in the supine position and secured to the operating table. After skin disinfection, an incision was made along the midline of the neck under stereomicroscopy. The left common carotid artery (CCA), internal carotid artery (ICA), and external carotid artery (ECA) were carefully separated, avoiding vagus nerve injury. Then, the ECA was severed and turned over to keep it as straight as possible with the CCA, which was then clamped with an arterial clip, and then a small cut was made in the ECA with ophthalmic scissors. After disinfection, the suture was inserted into the ICA. If there was slight resistance, it meant that the suture reached the beginning of the middle cerebral artery, and the insertion depth was approximately 20±2 mm (from the CCA bifurcation), as shown in Figure 2. The arterial clamp at the CCA was removed after the suture was fixed. After 2 h, the suture was removed, the ECA was ligated, the mid-neck incision was sutured and disinfected, and finally, the rat was returned to the cage and awakened. The same steps were followed in the control group, except that no suturing was performed.

### Quality control of high-resolution laser Doppler

**Figure 3:**
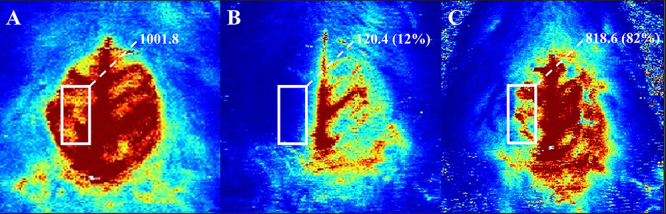
Representative images of the cerebral blood flow (CBF) monitoring by high-resolution laser Doppler indicated as the value in the white boxes. **A**, Baseline CBF in the middle cerebral artery. **B**, CBF is significantly decreased after inserting the suture on the ipsilateral side. **C**, Partial recovery of CBF on the ipsilateral side 24 h after reperfusion. The numbers indicate CBF in mL·100 g^-1^·min^-1^.

According to the Stroke Therapy Academic Industry Roundtable principles, cerebral blood flow (CBF) monitoring should be performed during stroke modeling procedures (17). Therefore, all rats included in the experiment were monitored for CBF to ensure the success of modeling. Rats were placed in the prone position and secured to the operating table after anesthesia. After disinfection of the spare skin, the scalp was cut along the midline, and the cranial periosteum was corroded with 3% hydrogen peroxide until the skull was exposed. Regional baseline CBF was measured by high-resolution laser Doppler (Moor lDI2-HIR, Moor Instruments, UK), as shown in Figure 3A. After inserting the suture, CBF decreased to <30% of the baseline blood flow, indicating successful modeling (Figure 3B). Successful reperfusion was indicated by a recovery of CBF to >70% of the baseline blood flow after removal of the suture on the ipsilateral side (Figure 3C).

### TTC staining

Rats were deeply anesthetized and then sacrificed by decapitation 24 h after reperfusion. Brain tissue was carefully dissected, and then the olfactory bulb and cerebellar brainstem were removed. The remaining brain tissue was cut into 5 coronal slices with an average thickness of 2 mm. The brain slices were spread flat on the bottom of a dish, totally immersed in a 2% TTC staining solution (Sigma-Aldrich, USA), and then incubated at 37°C for 30 min in the dark.

### Sampling the ischemic border zone

**Figure 4:**
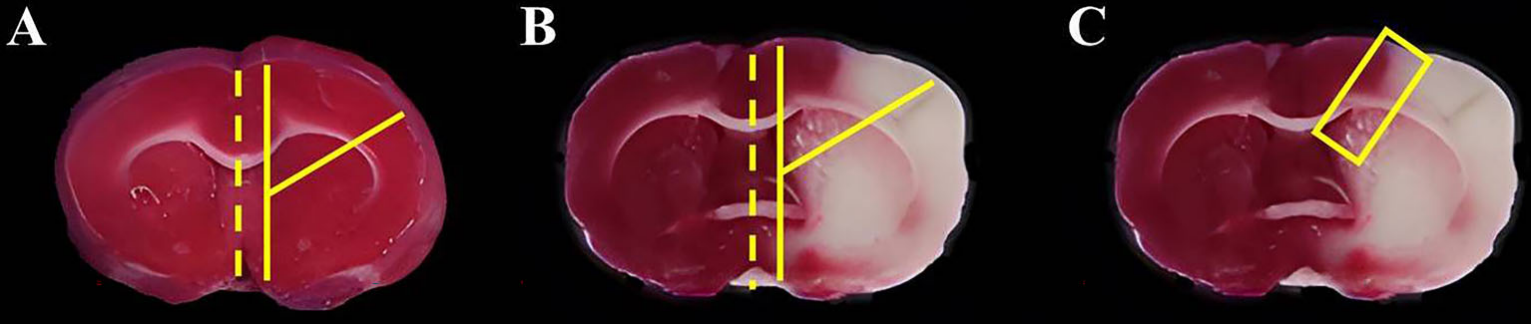
Sampling after TTC staining. **A**, Sampling of the control group by the two o’clock method. **B**, Traditional two o'clock method after TTC staining. **C**, Our proposed rectangular method in the modified group. The two o'clock method in panel A was performed without TTC staining. TTC: 2,3,5-triphenyl tetrazolium chloride.

After TTC staining, a coronal slice was taken at the level of the striatum. In the control group, the wedge-shaped brain tissue to be examined was obtained by the two o'clock method (Figure 4A). In the traditional group, the ischemic border zone of the ipsilateral side was obtained by the two o'clock method after TTC staining (Figure 4B); this group underwent the same sampling procedure as the control group. In the modified group, the ischemic border zone of the ipsilateral side was obtained by the rectangular method, as shown in Figure 4C. The upper border of the sampling area was taken as the edge of the cortex. The border of the red- and white-dyed TTC staining was the center line with a distance of 1 mm on each side, forming a rectangle of 2×5 mm. After sampling the ischemic border zone, the brain tissue was stored in a refrigerator at -80°C.

### Tissue protein concentration and western blot analysis

Frozen brain tissue was weighed, and an enhanced RIPA lysate (Boster, China) and PMSF protease inhibitor (Boster) were added. After full grinding, tissue homogenates were lysed on ice for 30 min, followed by 1000 *g* centrifugation at 4°C for 10 min. The supernatant was collected as the target proteins. The target protein concentration was determined according to the instructions of the BCA Protein Concentration Assay Kit (Boster).

A 10% separating gel and 5% stacking gel were prepared according to the SDS-PAGE gel Preparation Kit (Solarbio, China). Electrophoresis was performed using an electrophoresis apparatus (Bio-Rad, USA); the initial voltage was set at 80 V, and then it was increased to 120 V when bromophenol blue entered the separating gel. The duration of electrophoresis was 90 min. The membrane was then transferred using a semi-dry membrane transfer device (Bio-Rad) with the membrane transfer parameters of 16 V for 25 min. After transfer, the protein membrane was blocked in 5% non-fat milk (Boster). The following antibodies were diluted using a western blot-specific antibody dilution (Boster): rabbit polyclonal anti-superoxide dismutase 2 (SOD2, 1:1000, Abclonal, USA), rabbit polyclonal anti-cysteinyl aspartate specific proteinase 3 (caspase-3, 1:1000, Abclonal), rabbit polyclonal anti-caspase-9 (1:1000, Abclonal), rabbit polyclonal anti-heat shock protein 70 (HSP70, 1:1000, Abclonal), rabbit monoclonal anti-beta actin (1:50000, Abclonal), rabbit monoclonal anti-glyceraldehyde-3-phosphate dehydrogenase (1:5000, Abclonal), and goat anti-rabbit immunoglobulin G horseradish peroxidase (1:5000, Abclonal). The protein membrane was successively immersed in the configured primary and secondary antibodies. The luminescent solution was then prepared according to the instructions of the ultrasensitive luminescent substrate kit. An exposure instrument (Bio-Rad) was used for exposure, and the band was analyzed semiquantitatively using ImageJ software (National Institutes of Health, USA).

### Statistical analysis

Quantitative data are reported as means±SD. Multiple comparisons were performed using one-way analysis of variance (ANOVA) to determine significant differences between groups. Changes were identified as significant if the P value was less than 0.05. The SNK-q test was used for multiple comparisons. GraphPad Prism 9.0 software (GraphPad Software, Inc., USA) was used for data analysis. Statistical significance was set at P<0.05.

## Results

**Figure 5:**
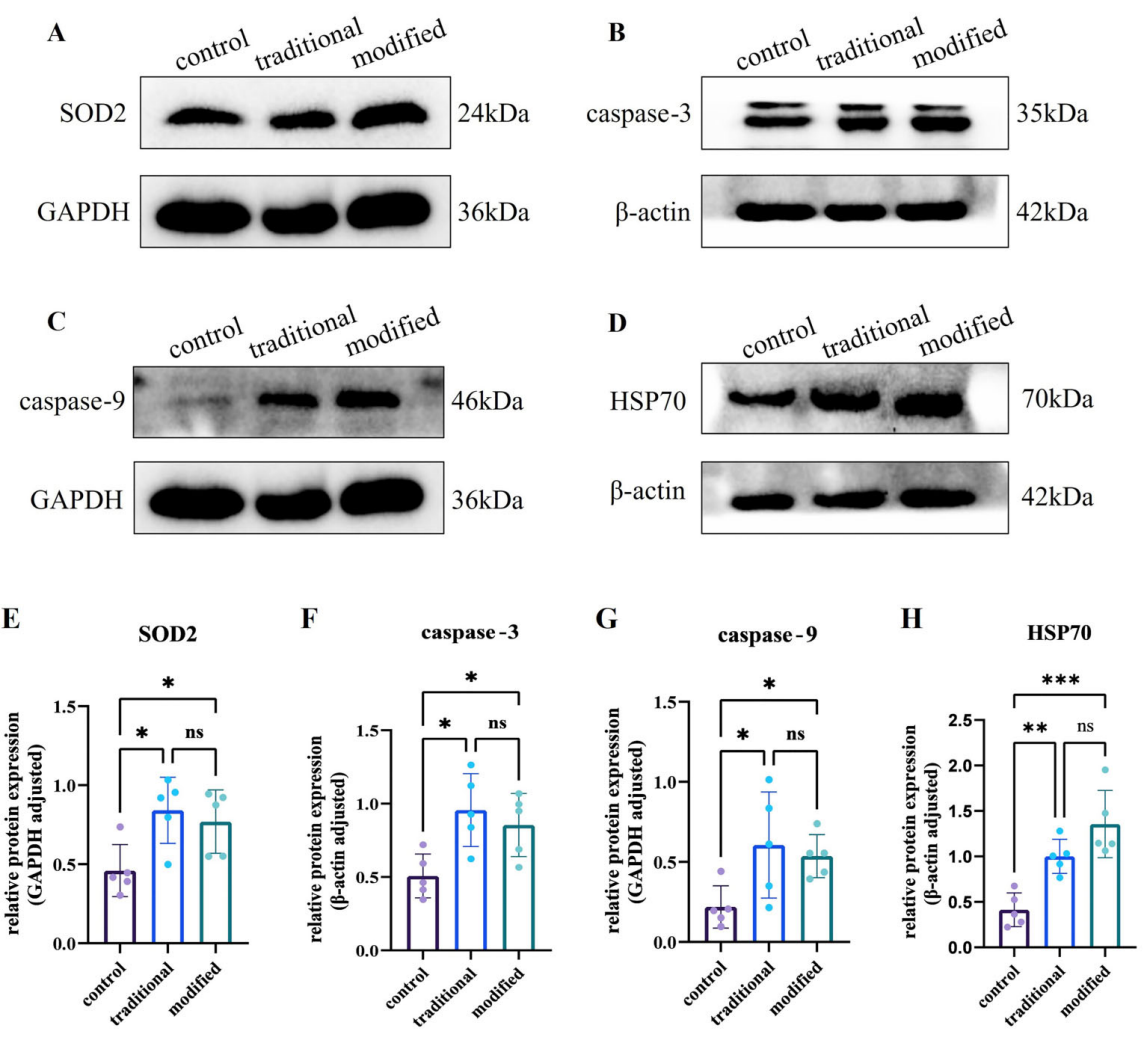
Western blot analysis of SOD2 (**A**), caspase-3 (**B**), caspase-9 (**C**), and Hsp70 (**D**) protein expression. **E**-**H**, Protein levels were measured by densitometric analysis. The data are reported as the signal intensity of the target protein bands relative to the internal reference bands. SOD2 and caspase-9 were electrophoresed at the same time, as were caspase-3 and HSP70. Therefore, some internal references can be shared. The lower band of caspase-3 is the target protein, which is closest to the molecular weight of 35 kDa. Data are reported as means±SD. *P<0.05, **P<0.01, ***P<0.001, ns: not significant (ANOVA). Groups: control: two o'clock method; traditional: two o'clock method after TTC staining; modified: rectangular method after TTC staining. SOD2: superoxide dismutase 2; HSP70: heat shock protein 70; caspase-3: cysteinyl aspartate specific proteinase 3; caspase-9: cysteinyl aspartate specific proteinase 9; TTC: triphenyl tetrazolium.

The protein levels of SOD2, caspase-3, caspase-9, and HSP70 in each group were detected by western blot analysis, and the signal intensity of the bands was quantified by ImageJ. As shown in Figure 5, all biomarker expressions were increased in the traditional and modified groups compared to their own control groups. There were no significant differences in the expressions of SOD2, caspase-3, caspase-9, and HSP70 between the traditional and modified groups.

## Discussion

In 1992, Memezawa et al. (18) found that perfusion in the fronto-parietal cortical motor area and medial caudate nucleus area was better than that in the fronto-parietal cortical sensory area and lateral caudate nucleus area in a rat middle cerebral artery occlusion model. Based on this study, Ashwal et al. (2) proposed the two o'clock method in 1998, which was the most widely used method due to its ease of use. However, due to individual differences between rats and different suture material, suture diameter, suture insertion depth, and ischemic time (19,20), there was a different infarct core and ischemic border zone (21). Unfortunately, most researchers do not perform TTC staining when using this method to obtain brain tissue from the ischemic border zone, which means that they cannot see the infarct during sampling. This makes it difficult to accurately assess the location and size of the infarct.

Recently, some researchers modified the two o'clock method (22-24). The diagonal method and parallel line method have been favored by some researchers in recent years. Both methods require localization based on TTC staining, and the principle of these methods is to take half of the sample from the red tissue and half from the white tissue after TTC staining. However, the ischemic border zone obtained by the diagonal method is too small to complete the subsequent experimental operation. The application of the parallel line method is limited since it may only be used in the cerebral tissue slices where the infarct is confined to the cortex. Our modified rectangular method may overcome the limitations of the diagonal method and parallel line method; that is, the brain tissue after TTC staining can be used to obtain the ischemic border zone without considering the location and size of the infarct. As long as the boundary between red and white tissue can be confirmed after TTC staining, the stable ischemic border zone can be obtained, which may greatly improve the credibility of the experimental results. Moreover, TTC immersion staining can be used as a reliable marker of cerebral ischemic injury with a survival time of 24 h after cerebral ischemia (25).

Previous studies have shown that there is no influence on the following experimental operations after TTC staining: western blot analysis (26), polymerase chain reaction (27), and immunohistochemical analysis (28). Our study validated the reliability of the rectangular method with the ischemic biomarkers by western blot analysis. As aforementioned, the expressions of all biomarkers in the ischemic border zone obtained by the two o'clock method and rectangular method were significantly increased compared with those in the control group. There was no significant difference in the expressions of markers in the ischemic border zone obtained by these two methods.

It is worth noting that the “ischemic penumbra” has been similarly described in previous studies with the three common traditional methods, but that does not justify its current use. This may lead to more confusion about its definition. For this paper, the only accurate description would be ischemic border zone, which differs from the absolutely precise ischemic penumbra as assessed by clinical multimodal imaging (29). In clinical practice, the definition of the penumbra is salvageable tissue adjacent to the ischemic core that does not yet show signs of cytotoxic edema but is metabolically suffering from hypoperfusion (30). However, the ischemic border zone in this study was defined as the area around the infarct core, in which markers such as SOD2, caspase-3, caspase-9, and HSP70 were significantly increased. Moreover, the above biomarkers were based only on sensitive indicators in the cerebral ischemic border zone from previously published studies, which can only indicate the comparability of the rectangular method, and does not adequately reflect the pathophysiology of the ischemic penumbra (31). In addition, the rectangular method requires TTC guidance. Even though some studies have shown that TTC staining has no effect on the subsequent experimental operations, pre-experiments should be conducted before adopting this method to determine whether it has an effect on the researcher-specific operations. Of note, when the infarct area is very small and close to the cortical edge, the rectangular method may not be able to obtain a sufficient area of 2×5 mm.

The rectangular method proposed herein is based on a modification of the diagonal method and parallel line method, and it could provide a directly observable infarct borderline and a sufficient sampling area for subsequent experimental operations regardless of the cerebral infarct location. The ischemic border zone biomarkers (SOD2, caspase-3, caspase-9, and HSP70) demonstrated the reliability and reproducibility of the rectangular method, which may facilitate inter-laboratory comparisons.
